# Assessing expected utility and profitability to support decision-making for disease control strategies in ornamental heather production

**DOI:** 10.1007/s11119-022-09909-z

**Published:** 2022-05-22

**Authors:** Marius Ruett, Tobias Dalhaus, Cory Whitney, Eike Luedeling

**Affiliations:** 1 INRES-Horticultural Sciences, University of Bonn, Auf dem Hügel 6, 53121 Bonn, Germany; 2 Business Economics Group, Wageningen University and Research, Hollandseweg 1, 6706 KN Wageningen, Netherlands; 3 Center of Development Research (ZEF), University of Bonn, Genscherallee 3, 53113 Bonn, Germany

**Keywords:** Decision support, Model simulation, Risk preference, Precision agriculture, Sensor application, Fungicide

## Abstract

Many farmers hesitate to adopt new management strategies with actual or perceived risks and uncertainties. Especially in ornamental plant production, farmers often stick to current production strategies to avoid the risk of economically harmful plant losses, even though they may recognize the need to optimize farm management. This work focused on the economically important and little-researched production system of ornamental heather (*Calluna vulgaris*) to help farmers find appropriate measures to sustainably improve resource use, plant quality, and profitability despite existing risks. Probabilistic cost-benefit analysis was applied to simulate alternative disease monitoring strategies. The outcomes for more intensive visual monitoring, as well as sensor-based monitoring using hyperspectral imaging were simulated. Based on the results of the probabilistic cost-benefit analysis, the expected utility of the alternative strategies was assessed as a function of the farmer’s level of risk aversion. The analysis of expected utility indicated that heather production is generally risky. Concerning the alternative strategies, more intensive visual monitoring provides the highest utility for farmers for almost all levels of risk aversion compared to all other strategies. Results of the probabilistic cost-benefit analysis indicated that more intensive visual monitoring increases net benefits in 68% of the simulated cases. The application of sensor-based monitoring leads to negative economic outcomes in 85% of the simulated cases. This research approach is widely applicable to predict the impacts of new management strategies in precision agriculture. The methodology can be used to provide farmers in other data-scarce production systems with concrete recommendations that account for uncertainties and risks.

**Supplementary information:**

The online version contains supplementary material available at 10.1007/s11119-022-09909-z.

## Introduction

Ornamental plant production systems are of considerable economic importance in Europe. Germany features the largest market for ornamental plants, with sales of more than 8.9 billion euros at retail prices in 2019 (Zentralverband Gartenbau e.V., 2019). For producers of ornamental plants, control of plant quality is critical for economic success. Frequent prophylactic pesticide treatments are the first choice for maintaining plant quality, as such treatments currently represent the most reliable and most cost-effective strategy (Shtienberg, 2013).

Frequent fungicide treatments are standard practice in ornamental heather (*Calluna vulgaris*) cultivation, a production system that requires a high level of specialization and expertise on the part of the growers. In an effort to reduce their reliance on high rates of fungicide applications, heather farmers are currently searching for alternative ways to control plant quality while reducing fungicide use.

The outcomes of alternative farming practices can be predicted using decision support systems that consider all important variables that matter in complex agricultural production (Luedeling & Shepherd, 2016). In general, decision support systems aim to assist a decision-maker by providing information that is relevant for a given problem so that actionable solutions can be delineated (Burstein & Carlsson, 2008). Decision support systems can be based on different methods and use various modeling approaches. For instance, supervised learning models like support vector machines (SVM) have been able to provide decision support by enabling early detection of fungal disease symptoms on sugar beet leaves (Rumpf et al., 2010). Using an argumentation-based approach, Thomopoulos et al. (2015) developed a reverse engineering method (by first setting the goal) to support decision-making in agricultural food chains regarding the impacts of production and storage on food quality. Winter chill models have been applied to predict how climate change scenarios might influence deciduous fruit production and to support farmers’ adaptation decisions (Fernandez et al., 2020). Based on multiple survey data, Wang et al. (2012) forecasted yearly crop yields using a Bayesian hierarchical model as the basis of a decision support system. Bayesian networks have been used to support decision-making in agricultural development contexts, including application of expert knowledge elicitation (EKE) approaches to consider existing risks (Whitney et al., 2018; Yet et al., 2016). EKE-based decision support was also provided by MacMillan & Marshall (2006), who applied the Delphi method to guide wildlife conservation management.

Involving experts through collaborative modeling approaches is crucial for development of credible decision support systems, and it allows accounting for agricultural risks (Oliver et al., 2012). Probabilistic model simulations using collaborative workshops to generate input data based on expert estimates have been applied in several studies to support stakeholders’ risk management in production systems around the globe (Do et al., 2020; Rosenstock et al., 2014; Tamba et al., 2017; Wafula et al., 2018; Yigzaw et al., 2019). In horticultural production, similar approaches have been applied to predict the outcome of risk management strategies in sweet cherry production (Rojas et al., 2021). Apart from the mentioned decision support approaches, life cycle assessments combined with statistical risk assessment approaches constitute another digital tool that allows formulating recommendations for horticultural producers (Mouron et al., 2006). The literature confirms that digital risk management tools such as decision support systems are able to provide farmers with essential information to cope with risks. However, little literature is available on the evaluation of digital risk management tools in horticulture, although the potential of using such tools in combination with expert knowledge to provide decision support in horticulture has been recognized decades ago by Gary et al. (1998).

Alternative strategies to control plant quality while reducing fungicide use in heather production may incur risks of financial losses due to additional costs and unclear benefits. Heather farmers are exposed to a high risk of financial losses because plants are genetically similar or even identical, and the occurrence of fungal infections can be very dynamic, quickly leading to devastating plant losses (Ruett et al., 2020). Farmers may therefore hesitate to change their behavior, even if such a change holds a high chance of increasing their income. Distributions of likely outcomes produced by probabilistic analyses can indicate the likelihood of net benefits from adopting new strategies (Rojas et al., 2021), but they do not account for farmers’ individual risk preferences. An analysis of expected utility can deliver decision recommendations for different levels of risk aversion on the part of a decision-maker by transforming probabilistic estimates of possible outcomes into an expected utility value using a utility function (Chavas, 2004; Hardaker et al., 2015). The concavity or convexity of the utility function indicates individual risk preference profiles, ranging from ‘risk-taking, over ‘risk-neutral’, to ‘risk-averse’. Analysis of expected utility can therefore deliver detailed decision support that accounts for the decision-maker’s risk preferences (Schaub et al., 2020). The expected utility of disease control strategies and the level of risk aversion of heather farmers have not yet been investigated. Incorporating risk perceptions and risk preferences into decision support systems is promising and might increase the utility of disease management for agricultural production systems (Gent et al., 2011).

Ornamental heather production systems are data- and research-poor working environments, where economic success mostly depends on individual experience. The overall aim of the research project INRUGA (see funding) is to support heather farmers by developing collaborative Decision Analysis tools to optimize production conditions and make systems more resilient. Additionally, the project aims to test the potential of sensor technologies to analyze the plant health status of heather plants.

From 2018 to 2019, a first decision model was developed together with heather farmers, fungicide experts, experimental trial managers, and horticultural scientists. The collaborative work aimed to incorporate all existing knowledge into a decision model to simulate the heather production process. The model allowed exploration of the potential of reduced prophylactic fungicide applications and more intensive visual monitoring of plant health status in heather production (Ruett et al., 2020). Results indicated that just reducing prophylactic fungicide treatments is likely to lead to a lower Partial Farm Budget compared to standard cultivation practices. More intensive visual monitoring was likely to increase the Partial Farm Budget compared to standard cultivation practices. Expected Value of Perfect Information (EVPI) Analysis revealed that the respective costs and benefits represent the main uncertainties for implementation of more intensive visual monitoring (Ruett et al., 2020). This evaluation indicated the need for a cost-benefit analysis of more intensive visual monitoring to gain sufficient confidence in this strategy to recommend it to farmers.

Monitoring can not only be performed visually by humans but also through the use of sensors, which enable efficient phenotyping of plants and support the detection and investigation of disease patterns (Mahlein et al., 2019). Hyperspectral imaging with optical sensors is a proven tool to detect abiotic stress symptoms (Behmann et al., 2014) and to predict plant compounds such as water, nitrogen, and pigment contents in plants (Ge et al., 2016). Hyperspectral imaging can also detect biotic stress symptoms induced by fungal plant pathogens (Bohnenkamp et al., 2019). In ornamental plant production, sensor-based precision agriculture technologies have contributed to risk management by detecting fungal diseases using thermal sensors (Gomez, 2014) and digital cameras (Wijekoon et al., 2008). Virus diseases have been identified using multispectral cameras (Polder et al., 2014). Sensor-based approaches have also been applied to heathers, but mainly aimed to analyze flowering phenology (Neumann et al., 2020) and to assess plant compounds (Mac Arthur & Malthus, 2012) rather than focusing on managing production risks. The heather farmers involved in the INRUGA project expressed interest in finding out how well hyperspectral sensors could monitor plant stress, since such tools might enable them to efficiently control plant quality. To assess the potential of hyperspectral sensor monitoring for farmers in ornamental heather production, hyperspectral imaging was applied in a heather production facility. Heather plants were classified according to their health status, based on their spectral signatures. Plants were successfully classified as ‘healthy’ or ‘stressed’ with an accuracy of 98.1%, using a Partial Least Squares regression (PLSR) model, which demonstrated the technical feasibility of hyperspectral sensor monitoring for heather production (Ruett et al., 2022). Since technical feasibility alone does not necessarily correlate with economic viability, Ruett et al., (2022) recommended a detailed cost-benefit analysis that considers the costs incurred in conducting hyperspectral measurements under realistic production conditions.

The present work aims to synthesize the results of model-based assessment of more intensive monitoring (Ruett et al., 2020) and the potential of hyperspectral imaging in heather production (Ruett et al., 2022) in the context of an actual farm-scale adoption decision. The central hypothesis is that the willingness of ornamental plant producers to replace prophylactic fungicide applications with sensor-based precision farming strategies depends on their attitude towards risk. The work presented here consists of a joint application of decision support tools containing collaborative group work approaches for elicitation of expert estimates, probabilistic cost-benefit assessment and analysis of expected utility, considering individual risk preferences. Limitations of established decision support systems, which often do not consider farmers’ individual risk preferences, can be overcome with this joint approach. Collaborative group work methods are used to engage experts on the respective agricultural production system to frame a conceptual impact model illustrating the expected impacts of innovative practices. Costs, benefits and probabilities are defined as input variables. After experts have been subjected to a so-called ‘calibration training’, the state of knowledge on the values of input variables to the conceptual model is quantified by experts in the form of probability distributions, which are approximated by confidence intervals. The bounds of these confidence intervals should contain all plausible values (considering 90% confidence intervals) that can appear for an input variable, e.g. the investment costs for new hardware. The conceptual model with quantified variables is then transferred into a digital environment and coded as a decision model to perform probabilistic simulations. Simulation results are then used to conduct sensitivity analyses (Partial Least Squares regression with calculation of the ‘Variable Importance in the Projection’, as well as analysis of the ‘Expected Value of Perfect Information’), and analysis of expected utility with a focus on farmers’ individual risk preferences.

This study contributes to precision agriculture research by evaluating the merits of sensor-based disease detection, which may hold potential for improving the spatial and temporal management of ornamental plant production. It also presents a strategy for integrating disparate pieces of information, including managers’ risk preferences, to provide customized, evidence-based support to agricultural decision-makers.

## Materials and methods

### Identification of principal stakeholders

The research approach taken in this study heavily relies on inputs from local stakeholders (Whitney et al., 2018). Stakeholder involvement and communication with subject-matter experts can greatly support the development of new promising interventions and generate comprehensive understanding of the key processes that drive the behavior of the target system (Young et al., 2013). Stakeholder involvement can also support practical implementation of research results (Mach et al., 2017).

Seven principal stakeholders (see acknowledgements) were involved in this study. They were well-versed in the implementation of new production strategies and capable of estimating implementation costs, as well as benefits for heather production. The expert team included four heather farmers, one economic consultant and two cultivation advisers with high technical understanding of heather growing strategies and broad knowledge of ornamental heather production and its risks. In the following sections, these principal stakeholders are referred to as ‘experts’.

### Development of the conceptual cost-benefit model

Conceptual models can be created through integrated modeling approaches that incorporate all available expert knowledge to provide a complete understanding of complex systems (Lanzanova et al., 2019). In developing such models, the choice of participatory methods needs to be aligned with the research objective and appropriate for the set of stakeholders involved (Villamor et al., 2014). Typically, collaborative group work approaches are used to create graphical impact pathways with all available experts in a series of face-to-face meetings and workshops (Whitney et al., 2018). Due to the COVID-19 pandemic, previous collaborative group work approaches needed to be extensively updated using tools like video conferences to comply with social-distancing rules. Video conferences were applied for the whole model development process between June and August of 2020.

Farmers’ current monitoring regime was modeled as the baseline scenario. Experts agreed on two main scenarios to be modeled as new strategies and evaluated through cost-benefit assessment and analysis of expected utility. These scenarios included more intensive visual monitoring and sensor-based monitoring. In the following sections, these strategies are referred to as ‘*Baseline*’, ‘*Improved*’ and ‘*Sensor*’, respectively.

#### 1) Baseline

Current regime of visual monitoring with occasional observations of plant health. Severely symptomatic plants are discarded. A low number of laboratory samples are used to identify plants hosting fungal pathogens. Fungicide treatments are carried out without a particular focus on the infection risk.

#### 2) Improved

Intensified visual monitoring with frequent observations. Even slightly symptomatic plants are discarded. A high number of laboratory samples is used to identify plants hosting fungal pathogens. Fungicide treatments are only carried out when farmers consider the infection risk to be high.

#### 3) Sensor

Sensor-based plant health monitoring using hyperspectral imaging. Initial investments in sensor acquisition and data processing are required. Similar to the *Improved* strategy, laboratory samples are used to identify plants infected with fungal pathogens, all symptomatic plants are discarded, and fungicide treatments are only carried out when farmers consider the infection risk high.

Together with experts, conceptual models were developed that contained all the costs and benefits that were considered important for each of the monitoring strategies, as well as the causal mechanisms through which costs and benefits arise. The overall merits of all strategies were quantified by calculating the Net Present Value (NPV). The NPV facilitates comparison of agricultural cultivation strategies. It accounts for the implications of initial investment needs, delayed profits and, in general, farmers’ time preference by discounting future costs and benefits through use of an investor-specific discount rate (Do et al., 2020). Following Do et al. (2020), the following equation was applied to calculate the NPV in the present work:

1 $$ NPV={-C}_{0}+ {\sum }_{i=1}^{t}\frac{{C}_{i}}{(1+r{)}^{i}}$$

${C}_{0}$ represents the establishment cost and ${C}_{i}$ the cash flow in year *i*. The discount rate is denoted by *r* and the time of simulation by *t*.

The collaboratively developed conceptual models expressed impact pathways for all candidate monitoring strategies. Experts continuously reviewed and updated the structure and content of the model sketches. As a last step, all resulting models were merged into one final graphical model and reviewed again by all experts, until everyone agreed on the final structure. This final conceptual model was then used as a framework to develop a mathematical model for cost-benefit simulation. The full mathematical model for cost-benefit simulation with detailed annotations, and all data generated in this study are available as supplementary materials in the following open-access repository: https://github.com/marruett/Supplementary_Ruett_Precision_Agriculture.

### Generating expert estimates

Cost-benefit analyses based on single, precise numbers usually fail to adequately consider risks and uncertainties (Luedeling et al., 2015). In contrast to risks, uncertainties in decision-making represent situations in which possible outcomes of decisions and the probability of their occurrence are unknown (Tversky & Fox, 1995). These shortcomings can be overcome by expressing variables using distributions, often specified by lower and upper bounds of confidence intervals that encompass all plausible values (Luedeling et al., 2015). Calculations with single, precise numbers can easily produce misleading results and give decision-makers a false sense of certainty about the prospects of particular investments. Decision Analysis approaches that work with distributions of all plausible values can overcome these limitations and facilitate decision support for stakeholders while incorporating the uncertainty of actual production conditions (Luedeling et al., 2015). This article reports on a cost-benefit analysis based on the principles of Decision Analysis (Howard & Abbas, 2015). This approach is rooted in the premise that it is usually impossible to obtain perfect information on all variables that are relevant for a given decision. It is however possible to estimate plausible ranges and distributions for such missing information based on available knowledge and expert judgement. Using such estimates can help overcome data availability constraints and allow for the assessment of the net benefits of management decisions, even in the absence of perfect data. The ability of experts to provide useful estimates that actually express the state of knowledge on particular variables can be enhanced through a process known as ‘calibration training’. From range estimates for all model input variables, the net benefits of particular intervention options can then be computed through probabilistic simulations (Tamba et al., 2021).

### Calibration training

To enhance the experts’ ability to estimate their uncertainty and to make them aware of cognitive biases, the whole expert team was subjected to calibration training. Depending on expertise and level of self-awareness, a person may over- or underestimate her ability to estimate variables. For the calibration training, sets of estimation questions were used, asking experts to specify their 90% confidence intervals for the answers in the form of a lower and an upper bound. After each round of estimation exercises, the participants compared their estimates with the correct solutions, indicated how they arrived at their intervals and reflected on how errors may have occurred. Through this reflection process, experts were confronted with their cognitive biases and potential biases in risk perception, motivating them to question the rigor of their quantitative assessment. Experts were taught how cognitive biases affect people’s judgment and informed about the ‘Dunning-Kruger effect’ (Kruger & Dunning, 1999) and ’anchoring effects’ (Tversky & Kahneman, 1974) to reduce estimation biases. In addition, experts were taught multiple techniques enabling them to provide accurate estimates of their own uncertainty through reasonable assumptions by using ‘Fermi questions’ (Tetlock & Gardner, 2015), imagining a project ‘PreMortem’ (Klein, 2008), and applying the ‘equivalent bet’ method (Hubbard, 2014).

After calibrating all experts, 90% confidence intervals were elicited from them as estimates for all the input variables of the model. After each variable estimation, experts again had to explain how they had arrived at their estimated intervals. All intervals were first estimated in individual meetings and then reviewed by all participants in plenary sessions to adjust intervals if necessary. The continuous updating allowed the estimates to be optimized to reflect the current state of knowledge (Table 1).

**Table 1:** Input variables of the model estimated by calibrated experts. Variable names, units, distribution types (posnorm = positive normal distribution, const = constant, tnorm = truncated normal distribution), lower bounds, upper bounds and short descriptions of the variables are provided

Variable	Unit	Distribution	Lower Bound	Upper Bound	Description
discount_rate	digit	posnorm	1	5	discount rate
var_CV	%	tnorm	5	15	desired coefficient of variation
n_years	years	const	10	10	years of production
production_area	ha	const	8	8	total area of the nursery field
chance_high_risk	%	tnorm	40	60	chance of a year presenting high-risk conditions
initial_investment_B	€	posnorm	100	200	initial investment costs to enable Baseline
initial_investment_I	€	posnorm	200	500	initial investment costs to enable Improved
initial_investment_S	€	posnorm	18,000	100,000	initial investment costs to enable Sensor
additional_investment_B	€	posnorm	50	100	additional costs to enable Baseline
additional_investment_I	€	posnorm	50	100	additional costs to enable Improved
additional_investment_S	€	posnorm	100	500	additional costs to enable Sensor
labor_costs_B	€	posnorm	100	1,500	monetary value reflecting the time spent by a person involved in maintaining Baseline
labor_costs_I	€	posnorm	500	3,500	monetary value reflecting the time spent by a person involved in maintaining Improved
labor_costs_S	€	posnorm	500	5,000	monetary value reflecting the time spent by a person involved in maintaining Sensor
post_processing_costs_B	€	const	0	0	costs of data post processing for Baseline
post_processing_costs_I	€	posnorm	0	0	costs of data post processing for Improved
post_processing_costs_S	€	posnorm	100	500	costs of data post processing for Sensor
sample_number_B	digit	posnorm	2	3	number of samples for Baseline
sample_number_I	digit	posnorm	2	15	number of samples for Improved
sample_number_S	digit	posnorm	2	6	number of samples for Sensor
lab_costs_per_sample	€	posnorm	15	70	laboratory costs per sample
plant_value_of_discarded_plant	€	posnorm	0.25	0.6	value of discarded plant
plant_value_of_A1_quality	€	posnorm	0.6	0.9	value of marketable plant with high quality
number_of_saved_high_quality_plants_B	digit	posnorm	500	6,000	number of high quality plants saved by Baseline
number_of_saved_high_quality_plants_I	digit	posnorm	2,000	18,000	number of high quality plants saved by Improved
number_of_saved_high_quality_plants_S	digit	posnorm	500	10,000	number of high quality plants saved by Sensor
adjustment_sample_size_B	%	tnorm	10	50	adjustment in sample size for Baseline
adjustment_sample_size_I	%	tnorm	10	30	adjustment in sample size for Improved
adjustment_sample_size_S	%	tnorm	10	30	adjustment in sample size for Sensor
resource_savings_B	€	posnorm	100	500	resource savings for Baseline
resource_savings_I	€	posnorm	500	1,000	resource savings for Improved
resource_savings_S	€	posnorm	500	1,000	resource savings for Sensor

### Probabilistic simulation

The final cost-benefit model was coded in the R programming language (R Development Core Team, 2021) as a probabilistic Monte Carlo simulation. The simulation was run 10,000 times, with each model run initialized with random draws for all variables based on the distributions specified by the experts, to compute distributions of plausible NPV outcomes for all management strategies.

Partial Least Squares (PLS) regression was applied to evaluate the sensitivity of model outputs to variation in input variables (Luedeling & Gassner, 2012), using the ‘Variable Importance in the Projection’ (VIP) metric to estimate the influence of uncertainty about input values (Farrés et al., 2015). Regression coefficients of the PLS model were interpreted to characterize the correlation of input variables with the NPV. Following Galindo-Prieto et al., (2014), the following PLS model was applied to calculate VIP scores:

2 $${VIP}_{PLS}=\sqrt{K \times \left(\frac{\left[{\sum }_{a=1}^{A}({W}_{a}^{2} \times {SSY}_{comp, a})\right]}{{SSY}_{cum}}\right)}$$

The VIP describes the importance of variables in a PLS model using components and therefore represents the weighted combination of overall components of the PLS weights ${W}_{a}^{2}$. ${SSY}_{comp, a}$ represents the sum of squares of the dependent variable $Y$ explained by component $a$. $A$ represents the total number of components and $K$ the total number of variables (Galindo-Prieto et al., 2014).

Value of Information Analysis was used to detect critical uncertainties that limit the ability of the Monte Carlo simulation to produce clear management recommendations. The EVPI metric estimates the monetary value of perfect information for each variable based on the potential of this information to prevent ‘wrong’ decisions, i.e. the selection of a decision option that proves sub-optimal after implementation. The EVPI can be interpreted as the amount of money that a farmer should be willing to pay in order to attain perfect information regarding the respective input variable (Hubbard, 2014). The following equation was used to calculate the EVPI:

3 $$EVPI=EV|PI-EMV$$

$EVPI$ is calculated as the difference between the expected value of the outcome variable $EV$ given accurate knowledge on the value the tested input variable will take (perfect information – $PI$) and the expected maximum value $EMV$ of the outcome variable given only knowledge about the probability distribution of the input variable (imperfect knowledge). All analyses were conducted with the decisionSupport package for R (Luedeling et al., 2021).

### Calculation of expected utility

Based on the probabilistic simulation of possible NPV outcomes, farmers can decide to change their monitoring strategies or keep the production practices they currently use. This study assumes that this decision-making process is determined by the utility that farmers would gain from the different monitoring strategies and that farmers select the strategy that provides the highest expected utility. The expected utility can be derived from the distribution of possible NPVs using a utility function. This utility function represents farmers’ individual risk preferences (from risk-taking to risk-averse) and translates each simulated outcome of the Decision Analysis into a utility value. The expected utility framework was used to simulate certainty equivalents for different levels of risk aversion and different monitoring practices. The decision rule is to identify the strategy that provides the highest certainty equivalent. The certainty equivalent is the amount of money (fixed income) farmers would need to be offered in order to switch from the risky investment to a fixed income. The certainty equivalent thus reflects an individual’s taste for risk, i.e. her risk preference. Depending on the risk preferences of farmers and the riskiness of the investment, the certainty equivalent is smaller, equal or greater than the expected value of the risky investment (Chavas, 2004). Risk-averse farmers have a positive willingness to pay (risk premium) to get rid of the risk included in the investment option. Thus, their certainty equivalent is lower than the expected value of the risky investment. Risk-neutral decision-makers do not consider risk in their decision-making and thus for them the certainty equivalent equals the expected value of the risky prospect. Risk-taking farmers have a negative willingness to pay, i.e. they would need an additional external payment to switch away from their risky investment. They thus have a negative risk premium and a certainty equivalent that is greater than the expected value of the investment (Hardaker et al., 2015).

This framework allowed assessing the different monitoring strategies and their corresponding NPV distributions through the lens of farmers along a risk preference gradient. Here, the procedure proposed by Antle (1983), Chavas (2004), and Finger (2013) was used to calculate the certainty equivalent for each of the NPV distributions.

4 $${CE}_{k}=E\left({NPV}_{k}\right)-{RP}_{k}$$

In Eq. (4), ${CE}_{k}$ denotes the certainty equivalent of monitoring strategy *k.* $E\left({NPV}_{k}\right)$ is the expected value of the probabilistic NPV distribution of monitoring strategy *k*. ${RP}_{k}$ is the corresponding risk premium, i.e. the willingness to pay to remove all the risk from the stochastic ${\tilde{NPV}}_{k}$ (the tilde above the NPV symbol indicates the stochastic nature of this parameter). ${RP}_{k}$ can then be approximated (Arrow-Pratt approximation) based on the variance ${\sigma }_{NPV,k}^{2}$ of the ${\tilde{NPV}}_{k}$ distribution, which holds only for normally distributed NPVs (Chavas, 2004):

5 $${RP}_{k}\approx \frac{1}{2}\bullet {r}_{a}\bullet {\sigma }_{NPV,k}^{2}$$

${r}_{a}$ constitutes farmers’ risk aversion (constant absolute risk aversion), i.e. the aversion against variance in the stochastic ${\tilde{NPV}}_{k}$. ${r}_{a}$ is based on a farmer’s individual utility function $U$ and can be calculated from the first and second derivative of the utility function as ${r}_{a} =-{U}^{\prime \prime} / {U}^{\prime}$. For $U$ an exponential utility function of the form $U\left({NPV}_{k}\right)=1-{e}^{-{r}_{a}{\bullet NPV}_{k}}$ was chosen, which is a common representation of farmers’ utility (Hardaker et al., 2015). Negative values of ${r}_{a}$ indicate risk-taking preferences, while positive values imply a risk-averse farmer. The certainty equivalent was determined for different risk preferences ranging from ${r}_{a}=$-0.1 to ${r}_{a}$=0.1.

To put a particular emphasis on downside risks and allow for non-normally distributed NPVs, Eq. (5) was adjusted and the variance of NPV ${\sigma }_{NPV,k}^{2}$ replaced by twice the semi-variance ${SV}_{k}$, which is the variance in below-average NPV realizations, as suggested by Conradt et al., (2015) for a sensitivity analysis. This leads to:

6 $${RP}_{down,k}\approx {r}_{a}{\bullet SV}_{k}$$

Approximating the risk premium based on the moments of the NPV distribution, i.e. the semi-variance, allowed to place a particular weight on downside risks in a comparison of the different monitoring strategies rather than deriving the utility values directly from the utility function. Since this study is interested in the certainty equivalent of the different monitoring strategies along different levels of decision-maker risk aversion, the ‘stochastic efficiency with respect to a function’ (SERF) approach proposed by Hardaker et al., (2004) was adopted. This allows for plotting the certainty equivalents obtained from the different monitoring strategies as a function of risk aversion. For a given level of risk aversion one can thus easily identify the one strategy that provides the highest certainty equivalent, i.e. the optimal strategy from the farmer’s perspective.

The analysis of expected utility thus translates probabilistic simulation results into certainty equivalents based on different levels of risk preference. The SERF approach then allows making clear decision recommendations for the most beneficial monitoring strategy.

Expected utility is a normative theory for decision making under risk given certain risk preferences. To avoid biased inference through subjective probability perceptions, the calibration training described above was applied to raise the objectivity of estimates. Other theories such as cumulative prospect theory (Tversky & Kahneman, 1992), rank dependent utility (Quiggin, 1991), and salience theory (Bordalo et al., 2012) might be better able to predict actual behavior, which is however outside the scope of this analysis. Risk preferences in expected utility theory are not considered irrational and methods exist to elicit preferences (Holt & Laury, 2002).

## Results

### Conceptual cost-benefit model

**Fig. 1:**
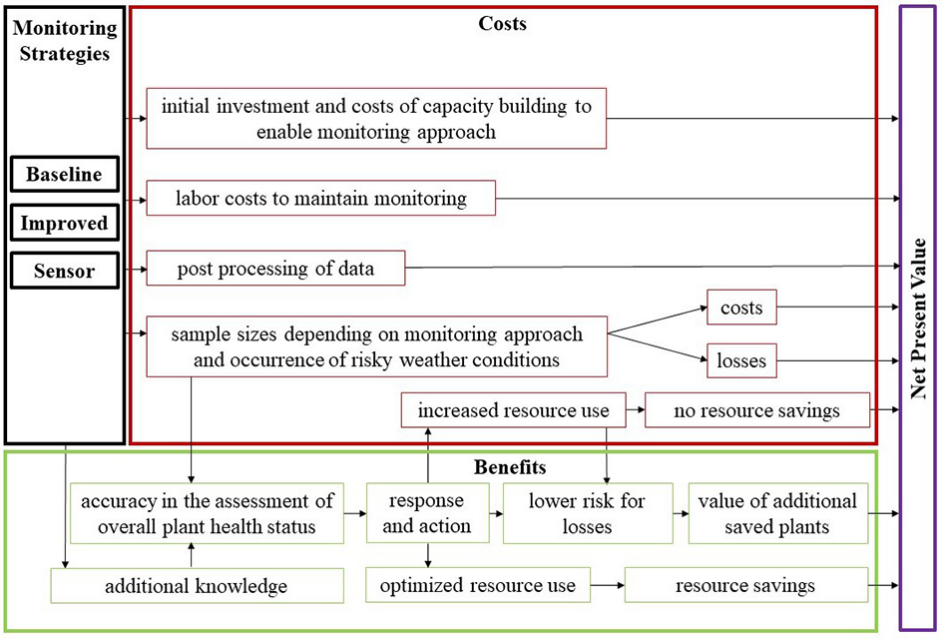
Conceptual cost-benefit model for monitoring strategies in commercial heather production. The different monitoring strategies are shown in black. The model output is quantified by the Net Present Value (purple). Benefits of the monitoring strategies are shown in green, costs in red

The conceptual model consisted of expert-identified costs and benefits that were expected to affect the Net Present Value (NPV) of the different monitoring strategies. Figure 1 illustrates the conceptual cost-benefit model for the monitoring strategies *Baseline*, *Improved*, and *Sensor*. The different monitoring strategies are shown by boxes with black outlines. The model output is quantified by the NPV (purple outline). Benefits of the monitoring strategies are framed in green, costs in red. These colors are also used for boxes within the cost and benefit categories, which stand for the various input variables. Arrows illustrate how the experts defined the impact of new monitoring strategies on associated costs and benefits, and thus on the NPV, in a commercial heather production system.

### Code description

The conceptual model was used as a blueprint to program the mathematical model. Monitoring strategies were simulated over a ten-year time period. Initial investments during the first year, which were deemed necessary for establishing a new monitoring approach (e.g. sensors, lamps, cameras, posters for the assignment of symptoms etc.), were taken into account. Maintenance costs required for all monitoring schemes were considered over the entire simulation period. All further costs and all benefits were calculated per unit area (based on 1 ha) and then multiplied by the estimated size of an average commercial heather production system in Germany (8 ha). Labor costs and post-processing costs for recording hyperspectral data were defined for each year of the simulation. Interannual variation was introduced for all costs and benefits to make the simulations more realistic. The sample number for laboratory analyses of fungal pathogens was defined separately for normal years and for high-risk years featuring a high frequency of high-humidity weather conditions that favor fungal infections. To date, no clear trend has emerged for the occurrence of high-risk years, which have so far occurred in roughly half of all years. This uncertainty was accounted for by estimating a 90% confidence interval for the chance of high-risk years between 40% and 60%. In normal-risk years, samples are taken less frequently because infection risks are expected to be lower. In high-risk years, more samples are taken and analyzed in laboratories to identify infected plants. This strategy can convey information about the spatial distribution of symptomatic plants within the production system. The costs of sampling and the value of discarded plants are added to the costs of each monitoring strategy.

The benefits of monitoring, which allows targeted disease prevention measures, were calculated within the production system. In normal-risk years, the additional knowledge about plant health status leads to resource savings, which are achieved when disease pressure is low and plant vitality is high. In high-risk years, monitoring approaches are expected to reduce plant losses and quality deficiencies by facilitating early removal of symptomatic plants and enabling targeted fungicide applications. Major resource savings are not achieved in high-risk years, but knowledge of the current plant conditions leads to increased reliability and predictability of production, which facilitates marketing and allows taking more high-quality plants to the point of sale.

Resource savings and a higher number of marketable, high-quality plants thus represent the potential benefits from improved monitoring strategies. The costs incurred were subtracted from the benefits in each year over the modeled time period and discounted to arrive at the NPV for each monitoring strategy. In the supplementary materials, the full code with all calculations of the cost-benefit model is provided as an R script.

### Projected outcomes of monitoring strategies

**Fig. 2:**
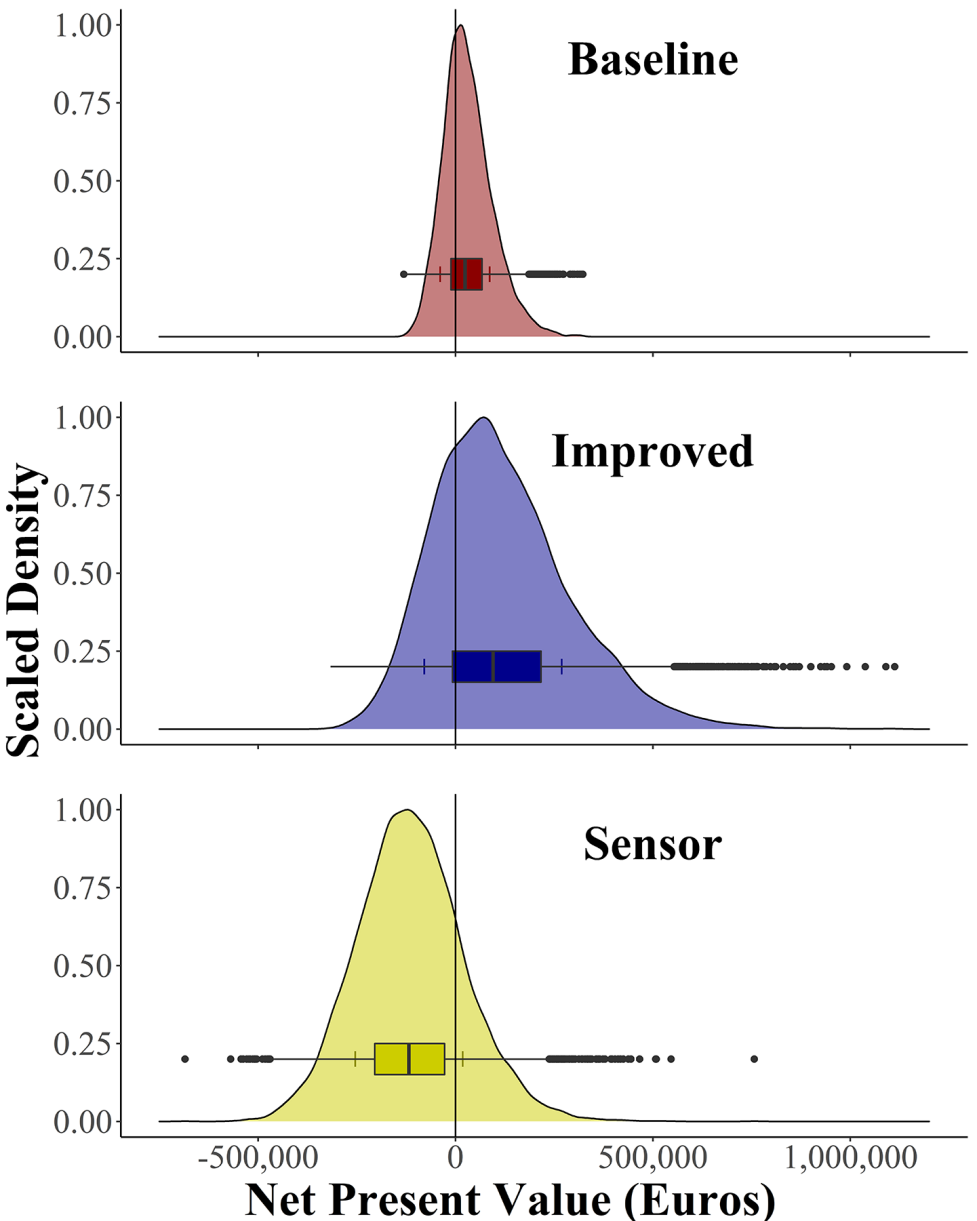
Probability density distributions (scaled density among 10,000 runs of a Monte Carlo simulation) of the Net Present Value (Euros) for three heather monitoring strategies. *Baseline*: Current visual monitoring regime. *Improved*: More intensive visual monitoring. *Sensor*: Sensor-based monitoring. The short vertical lines on the whiskers of the box plots indicate the standard deviation of the NPV for each strategy

The projected outcomes of monitoring strategies showed different shapes of predicted NPVs for *Baseline*, *Improved*, and *Sensor* based on an 8-ha heather production system (Fig. 2).

The modeled NPV for *Baseline* ranged from − 131,003 € to 322,594 €, with a probability of 67% of obtaining positive outcomes. For *Improved*, the NPV ranged from − 316,483 € to 1,112,458 €, with a chance of 73% of obtaining positive outcomes. The NPV of *Sensor* ranged from − 685,070 € to 756,778 €. The *Sensor* strategy was the only option with predominantly negative results, with 81% of the NPV distribution indicating a net loss.

### Analysis of expected utility

**Fig. 3:**
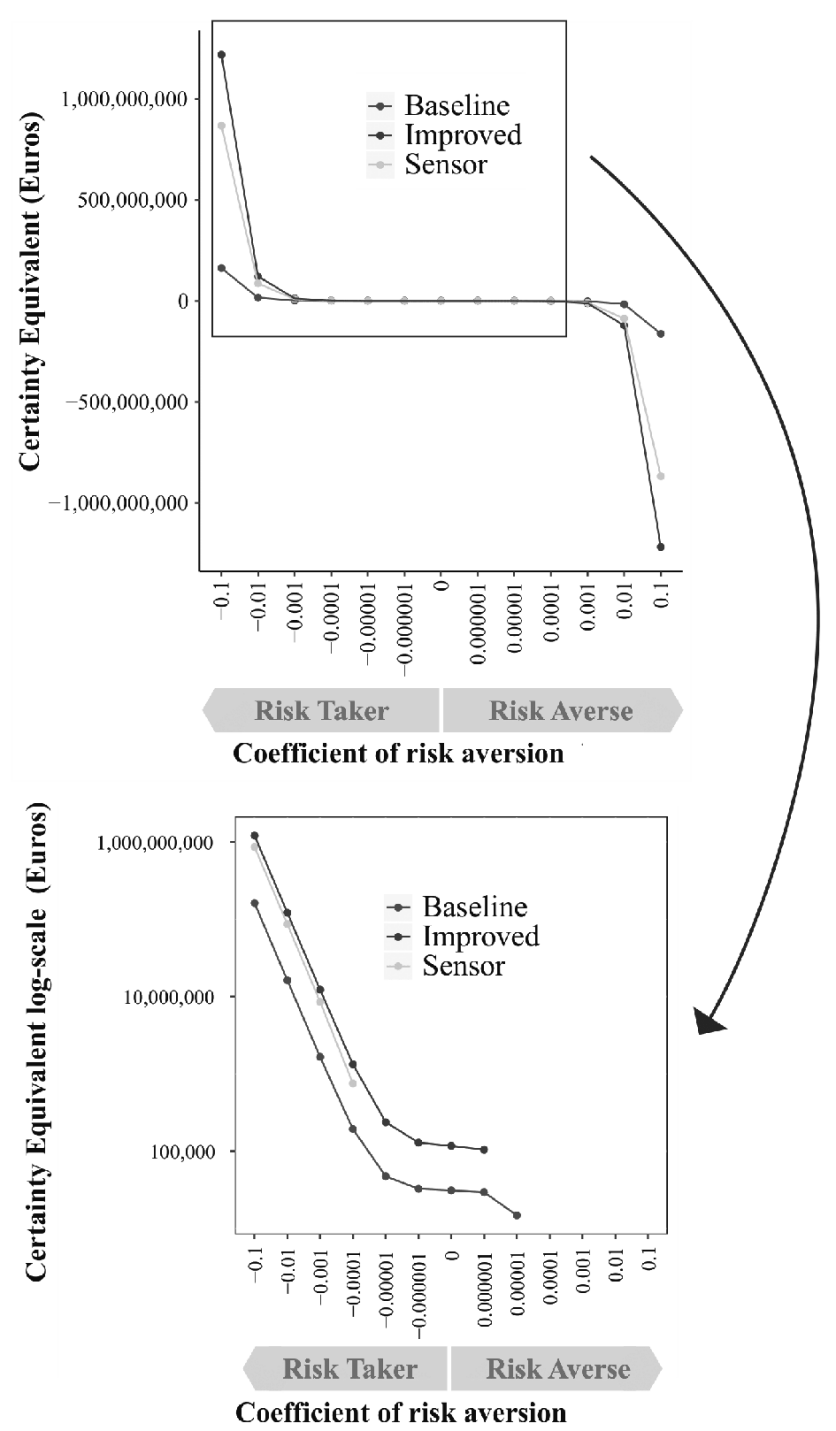
Stochastic efficiency with respect to a function (SERF) for monitoring strategies *Baseline*, *Improved*, and *Sensor.* In the upper figure the y-axis is shown on a normal scale, while the lower figure uses a log-scale to zoom into the positive parts of the SERF functions. In the latter case negative values are dropped from the illustration, because negative certainty equivalent values imply that decision-makers would not adopt an activity at all

The results of the analysis of expected utility were illustrated by plotting certainty equivalent values as functions of risk aversion for the different monitoring strategies (Fig. 3).

This illustration translates the probability distributions (shown in Fig. 2) into certainty equivalents for different risk preferences from risk-taking (coefficient of risk aversion < 0) over risk-neutral (coefficient of risk aversion = 0) to risk-averse (coefficient of risk aversion > 0). For a coefficient of risk aversion of 0, the certainty equivalents of the monitoring strategies are equal to the expected value of the NPV. The monitoring strategy that provides the highest certainty equivalent is the recommended strategy for a given level of risk preference. Results in the upper part of Fig. 3 show that for most of the risk-aversion preferences tested, all monitoring strategies result in negative certainty equivalents. These values imply that decision-makers would not adopt the activity at all. For risk-averse decision-makers, *Baseline* delivers low positive certainty equivalents in the risk-averse domain (for coefficients of risk aversion between 10^− 6^ and 10^− 5^). This suggests that heather growers in general tend to have rather risk-taking preferences. For risk-averse decision-makers, *Improved* shows an even lower positive certainty equivalent in the risk-averse domain (10^− 6^). For application of *Sensor*, positive certainty equivalents can be observed only in the risk-taking domain (from − 10^− 4^ downwards). This implies that from an expected utility point of view, heather production in general and all monitoring strategies considered here are rather preferred by risk-taking decision-makers. They potentially deliver high upside outcomes, but they also involve a considerable risk of failure.

Comparing the three monitoring strategies, decision-makers with risk preferences ranging between − 10^− 1^ and 10^− 6^ would prefer *Improved* over *Baseline* and *Sensor*. This implies that increased visual inspection of plants to reduce fungicide spraying is beneficial for a wide range of risk preferences and that farmers with such preferences would adopt improved monitoring. Only decision-makers who are strongly risk-averse (~ 10^− 5^), would prefer *Baseline* over the other monitoring strategies. The sensor technology cannot outperform the other monitoring strategies for any of the risk preferences tested here.

### Projected outcomes of monitoring decisions

The stated monitoring strategies were compared with the baseline monitoring practice to determine the likelihood that adopting alternative monitoring strategies increases the net benefit for farmers. The outcome of *Improved* and *Sensor* were subtracted from *Baseline*, keeping similar risk factors (e.g. the distribution of high-risk and normal-risk years) to achieve a fair comparison of monitoring strategies. This allowed to formulate the following decisions:

***1) DoMoreVisual*** (*Improved* – *Baseline*): The decision to switch from the current visual monitoring regime to intensified visual monitoring.

***2) UseSensor*** (*Sensor* – *Baseline*): The decision to switch from the current visual monitoring regime to sensor-based monitoring.

**Fig. 4:**
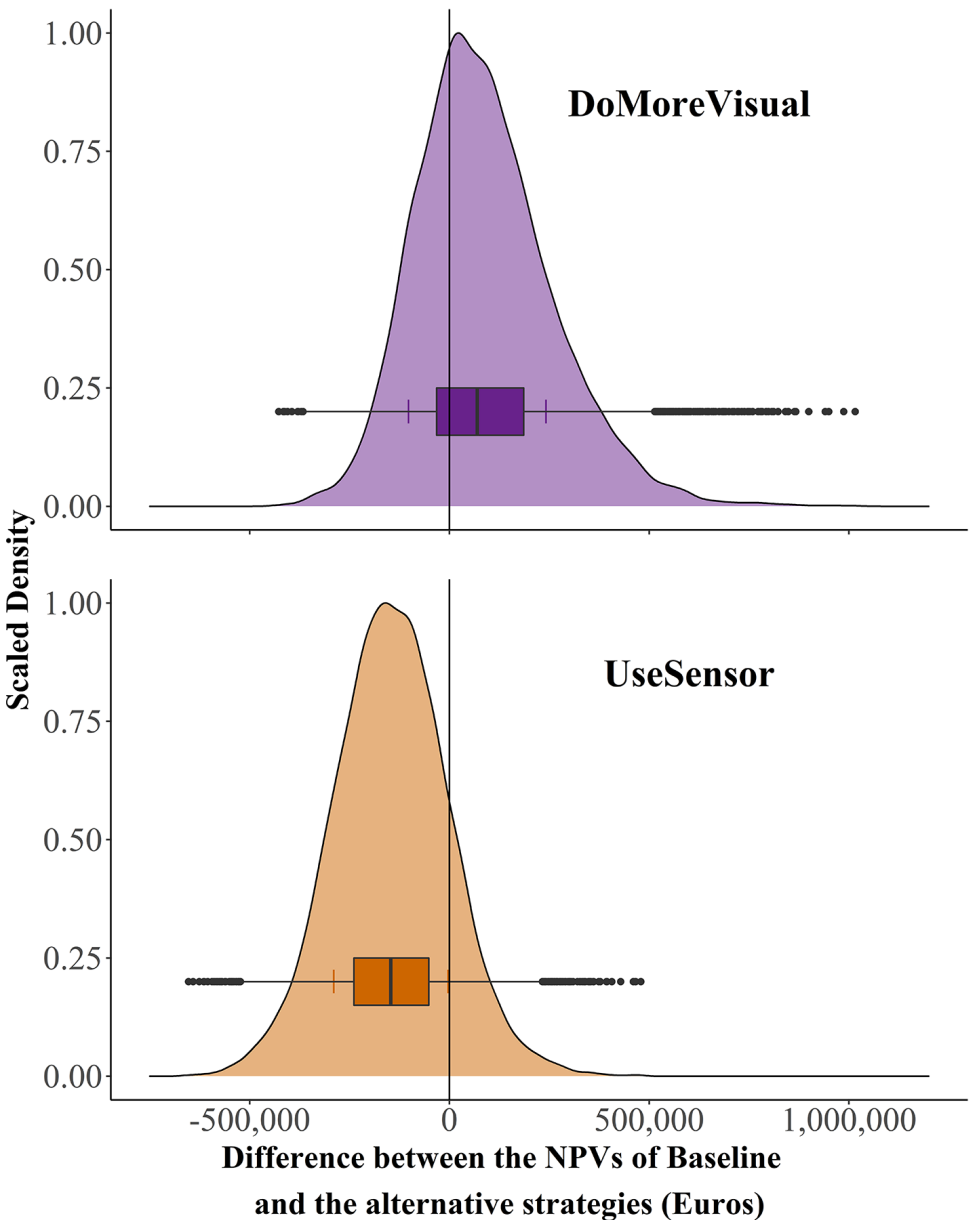
Probability density distributions (scaled density among 10,000 runs of a Monte Carlo simulation) of Net Present Values (Euros) for two heather monitoring decisions. *DoMoreVisual*: More intensive visual monitoring compared to the current monitoring regime. *UseSensor*: Sensor-based monitoring compared to the current monitoring regime. The vertical lines showing the color of the respective probability distribution reveal the standard deviation of the NPV for each decision

The projected outcomes (Fig. 4) of monitoring decisions indicated a high likelihood of 68% of achieving a positive NPV, with a mean NPV of 85,965 € for *DoMoreVisual*. The decision *UseSensor* showed an 85% likelihood of a negative NPV, with a mean projected NPV of -144,875 €.

### Important variables and uncertainties for monitoring decisions

**Fig. 5:**
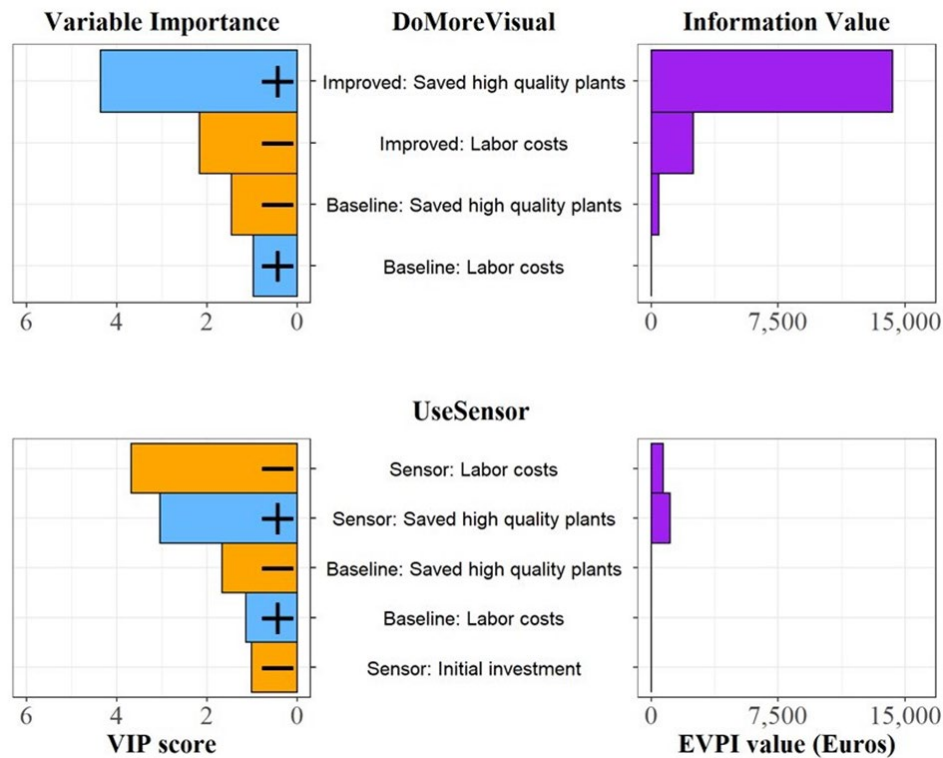
Variable Importance in the Projection (VIP scores) and Information Value (EVPI values) for the *DoMoreVisual* and *UseSensor* decisions. For the Variable Importance, only variables with VIP scores > 1 are shown. Variables that have a positive impact on the projected NPVs are represented by blue bars and a positive sign ‘+’, while those with a negative impact are represented by orange bars and a negative sign ‘-’

The results of the ‘Variable Importance in the Projection’ (VIP) and ‘Expected Value of Perfect Information’ (EVPI) metrics show the most important variables and the greatest uncertainties for the *DoMoreVisual* and *UseSensor* decisions (Fig. 5).

The highest-value variable in the *DoMoreVisual* decision was the number of high-quality plants saved due to *Improved* monitoring (EVPI = 14,274 €). Decision-makers would thus benefit from additional information on the number of plants that have increased quality because they use *Improved* monitoring rather than the *Baseline* strategy. This variable was also the main driver of the NPV for the *DoMoreVisual* decision, as indicated by a high VIP score.

For the *UseSensor* decision, high quality plants saved due to the sensor-based monitoring (EVPI = 1,096 €) and labor costs due to the use of sensor technology (EVPI = 695 €) represent the main uncertainties. The NPV for this decision was most sensitive to the labor costs of sensor-based monitoring (VIP = 3.68), which was negatively correlated with the NPV. The number of high-quality plants saved as a result of adopting the technology showed the second highest VIP score (VIP = 3.03).

## Discussion

Assessing the expected utility and profitability for the most promising monitoring strategies allowed supporting decision-making under uncertainty in the complex system of ornamental heather production. The outcome distributions of the strategies *Improved* and *Sensor* are wider compared to *Baseline*, highlighting the uncertainty when it comes to quantifying values for strategies that have not yet been widely applied (Fig. 2). Probability distributions of *DoMoreVisual* and *UseSensor* show that these decisions can lead to a wide range of possible outcomes with either negative or positive NPVs (Fig. 4). Given such wide outcome distributions, analyses of individual risk preferences, EVPI values and VIP scores are required for a more detailed assessment of the relative merits of the monitoring strategies. Risk-taking farmers are expected to be willing to apply more intensive visual monitoring to optimize their production system while sensor-based monitoring does not appear to be recommendable.

### Risk preferences of heather farmers

Heather production is an inherently risky business. The analysis of expected utility reveals that regardless of the selected monitoring strategy, heather production is mostly attractive to risk-taking decision-makers. The outcome distribution indicated that only decision-makers in the risk-averse domain (i.e. for risk aversion coefficients between 10^− 6^ and 10^− 5^) are likely to produce heather. Considering that a large share of European farmers appears to be risk-averse (Iyer et al., 2020), results might suggest that heather production is attractive to only a small share of the overall farmer population. Advancing monitoring strategies will require improved potential upside outcomes (e.g. by increasing plant quality and number of marketable plants), rather than cutting down on potential downside outcomes.

### Potential of more intensive visual monitoring

*DoMoreVisual* showed a high likelihood of positive NPVs (Fig. 4), with 68% of the NPV results located in the positive area. This implies that farmers who expend relatively little effort and money in the current monitoring regime (*Baseline)* would likely be able to increase their net benefits by adopting more intensive visual monitoring (*Improved*). *Improved* appears to facilitate safer production conditions, since it can raise the number of high-quality heather plants, which is one of the main benefits of the *DoMoreVisual* decision. Farmers and the environment would benefit from safer production conditions, which could be achieved by reducing the frequency of fungicide treatments whenever fungal infection risks are low. Although farmers do not need to apply fungicides when the infection risk is low (Bika et al., 2020), many farmers might prefer to use fungicides rather than expose their production system to the risk of harmful plant losses. In this regard, *Improved* represents a monitoring strategy that may make farmers reduce unnecessary fungicide applications, since frequent visual observations provide them with reliable and up-to-date information about the current health status of their plants.

The number of high-quality plants that can be produced due to more intensive monitoring not only registered a high VIP score; it also represents the greatest uncertainty according to the EVPI analysis (Fig. 5). Decision-makers would therefore benefit from additional information to reduce uncertainty regarding this variable.

Based on the results, risk-taking farmers who are concerned about environmental sustainability are willing to apply *Improved* monitoring to optimize their production system. This impression is supported by the analysis of expected utility, which indicates that risk-taking heather farmers aiming to maximize upside outcomes would prefer *Improved* over the *Baseline* and *Sensor* strategies (Fig. 3). Risk-averse farmers will probably continue to use frequent fungicide treatments using the *Baseline* strategy. Even if some farmers stick with current production methods, external influences such as customer demand for reduced fungicide treatments may encourage them to reconsider their practices at a later date (Shtienberg, 2013).

### Potential of sensor-based monitoring

For *UseSensor*, only 15% of all NPVs were located in the positive range (Fig. 4), indicating that the use of the technology is likely to decrease net benefits for heather farmers. To better inform the choice, decision-makers should be willing to invest in additional information on the labor costs involved in using a sensor and the effect of sensor-based monitoring on the number of marketable high-quality plants. The impact of sensor-based monitoring on the number of high-quality plants had a high EVPI within the *UseSensor* decision, followed by the labor costs of the strategy (Fig. 5).

Sensor-based monitoring through hyperspectral imaging has been identified as an effective approach for detecting plant diseases in greenhouses (Paulus & Mahlein, 2020) and agricultural fields (Bohnenkamp et al., 2019). The potential of hyperspectral imaging has been demonstrated for early detection of plant-related stress symptoms (Behmann et al., 2014), and for plant reactions to fungal diseases (Kuska et al., 2015). Despite this high technical potential, most studies have not considered the labor and costs required to perform regular sensor measurements in agricultural production systems. For the model of this study, the VIP scores identify an economic bottleneck in the labor needed to apply sensing technology, which had the greatest impact on the outcome (Fig. 5). The results suggest that the labor requirement to conduct sensor measurements is currently too high, leading to a high likelihood that a switch to sensor-based monitoring would generate net losses.

Farmers explained that the purchase of hyperspectral sensors entails relatively high costs, which is one of the reasons why buying a sensor for practical applications has not been considered so far. Practical applications are becoming increasingly feasible, however, as sensor costs have dropped considerably in recent years (Zubler & Yoon, 2020). According to Stuart et al., (2020), prices for hyperspectral sensors can range between approximately 35,000 € and 116,000 €. The experts suggested that less expensive sensors of less than 20,000 € have already proven successful in detecting fungal infections in heather plants (Ruett et al., 2022). For the simulation, a wide range of price scenarios was covered using a range between 18,000 € and 100,000 € (see supplementary materials). Nevertheless, the initial sensor investment does not seem to have a strong impact on the outcome of the *UseSensor* decision according to the VIP score results (Fig. 5). Contrary to initial assumptions, the initial investment to buy a sensor had the lowest VIP among variables in the *UseSensor* model, with an EVPI value of zero indicating that this was not an important uncertainty in the decision-making process.

The labor cost of performing sensor measurements represents the variable that is mainly responsible for the high chance of negative outcomes for the *UseSensor* decision. A lower workload might increase the likelihood of the *Sensor* strategy generating net benefits. Using sensor-based monitoring in ornamental heather production does not appear recommendable based on the current state of knowledge.

## Conclusion and overall recommendation

Heather cultivation is an ornamental plant production system where farmers are exposed to risks and uncertainties that hamper the implementation of new strategies. Decision Analysis approaches and analysis of expected utility were used in this study to evaluate what types of farmers (risk-averse or risk-taking) are willing to implement the simulated monitoring strategies in practice, considering existing risks and uncertainties. Analysis of expected utility showed that more intensive visual monitoring is attractive to risk-taking farmers aiming to increase the chance of upside outcomes. Although results suggest that heather farmers are risk-takers, the risk-averse group of heather farmers would be more likely to stick with the occasional-monitoring strategy that is currently practiced. The use of sensor-based monitoring with hyperspectral imaging currently appears to be inhibited by high labor costs associated with sensor measurements. Further research into practical application of sensors should focus on the impact of sensor-based monitoring on the number of marketable plants. In this study detailed management recommendations were formulated for farmers in heather cultivation, a production system in which little research has been conducted so far. The recommendations are based on current knowledge of calibrated experts and allow customized advice to be given to farmers according to their individual risk preferences. This research makes an important contribution to precision agriculture by supporting farmers’ decision management, taking into account the overall variability of what might happen in the heather production system as new disease control strategies are applied. This research approach can be used for the analysis of other agricultural systems to provide farmers in data-scarce production systems with concrete recommendations, accounting for uncertainties and risks.

## Electronic supplementary material

Below is the link to the electronic supplementary material.

Supplementary Material 1

## Data Availability

Supplementary Materials: All data generated in this study are available in the following open access repository: https://github.com/marruett/Supplementary_Ruett_Precision_Agriculture and can be cited using the following DOI: https://zenodo.org/record/4780875#.YKjRbqgzY2w.

## Code Availability

The code is available in the above repository (see ‘Availability of data and material’).
