# Supplementary material for: Assessing expected utility and profitability to support decision-making for disease control strategies in ornamental heather production
Source: Precis Agric. 2022 May 22;23(5):1775–800. doi: 10.1007/s11119-022-09909-z (PMC9124294; doi:10.1007/s11119-022-09909-z)
Supplement: Supplementary file 1 — Supplementary Material 1 [file 11119_2022_9909_MOESM1_ESM.pdf]

Assessing expected utility and profitability to support decision-making for disease control strategies in ornamental heather production

Marius Ruett[1*], Tobias Dalhaus[2], Cory Whitney[1,3] and Eike Luedeling[1]

*Corresponding author. E-mail address: ruett@uni-bonn.de (M. Ruett)

ORCID of the authors:

0000-0002-2708-4035 (M. Ruett.); 0000-0001-5853-0942 (T. Dalhaus);

0000-0003-4988-4583 (C. Whitney); 0000-0002-7316-3631 (E. Luedeling)

[1] INRES-Horticultural Sciences, University of Bonn, Auf dem Hügel 6, 53121 Bonn, Germany

[2] Business Economics Group, University of Wageningen, Hollandseweg 1, 6706 KN Wageningen, Netherlands

[3] Center of Development Research (ZEF), University of Bonn, Genscherallee 3, 53113 Bonn, Germany

This supplementary is available under https://github.com/marruett/Supplementary_Ruett_Precision_Agriculture and outlines the Decision Analysis approaches used in Assessing expected utility and profitability to support decision-making for disease control strategies in ornamental heather production Ruett et al. (under review in Precision Agriculture). Here the input table and code is provided to allow for transparent reproduction of the model. To create and organize this supplementary file the packages devtools (Wickham et al., 2019b), knitr (Xie, 2019a), kableExtra (Zhu, 2019) and bookdown (Xie, 2019b) are used. All other applied R packages are cited and listed in the References.

Input table for assessment of expected utility and profitability to support decision-making in ornamental heather production

The input table Input_CBA.csv contains: Variable names, unit, distributions (posnorm = positive normal distribution, const = constant, tnorm_0_1= truncated normal distribution), lower bound, upper bound and description of all the input variables for the model. The abbreviation CBA refers to Cost Benefit Assessment.

Variable	Unit	Distribution	Lower Bound	Upper Bound	Description
discount_rate	digit	posnorm	1	5	discount rate
var_CV	%	tnorm	5	15	desired coefficient of variation
n_years	years	const	10	10	years of production
production_area	ha	const	8	8	total area of the nursery field
chance_high_risk	%	tnorm	40	60	chance of a year presenting high-risk conditions
initial_investment_B	€	posnorm	100	200	initial investment costs to enable Baseline
initial_investment_I	€	posnorm	200	500	initial investment costs to enable Improved
initial_investment_S	€	posnorm	18,000	100,000	initial investment costs to enable Sensor
additional_investment_B	€	posnorm	50	100	additional costs to enable Baseline
additional_investment_I	€	posnorm	50	100	additional costs to enable Improved
additional_investment_S	€	posnorm	100	500	additional costs to enable Sensor
labor_costs_B	€	posnorm	100	1,500	monetary value reflecting the time spent by a person involved in maintaining Baseline
labor_costs_I	€	posnorm	500	3,500	monetary value reflecting the time spent by a person involved in maintaining Improved
labor_costs_S	€	posnorm	500	5,000	monetary value reflecting the time spent by a person involved in maintaining Sensor
post_processing_costs_B	€	const	0	0	costs of data post processing for Baseline
post_processing_costs_I	€	posnorm	0	0	costs of data post processing for Improved
post_processing_costs_S	€	posnorm	100	500	costs of data post processing for Sensor
sample_number_B	digit	posnorm	2	3	number of samples for Baseline
sample_number_I	digit	posnorm	2	15	number of samples for Improved
sample_number_S	digit	posnorm	2	6	number of samples for Sensor
lab_costs_per_sample	€	posnorm	15	70	laboratory costs per sample
plant_value_of_discarded_plant	€	posnorm	0.25	0.6	value of discarded plant
plant_value_of_A1_quality	€	posnorm	0.6	0.9	value of marketable plant with high quality
number_of_saved_high_quality_plants_B	digit	posnorm	500	6,000	number of high quality plants saved by Baseline
number_of_saved_high_quality_plants_I	digit	posnorm	2,000	18,000	number of high quality plants saved by Improved
number_of_saved_high_quality_plants_S	digit	posnorm	500	10,000	number of high quality plants saved by Sensor
adjustment_sample_size_B	%	tnorm	10	50	adjustment in sample size for Baseline
adjustment_sample_size_I	%	tnorm	10	30	adjustment in sample size for Improved
adjustment_sample_size_S	%	tnorm	10	30	adjustment in sample size for Sensor
resource_savings_B	€	posnorm	100	500	resource savings for Baseline
resource_savings_I	€	posnorm	500	1,000	resource savings for Improved
resource_savings_S	€	posnorm	500	1,000	resource savings for Sensor

Implementing the model in R

Here the mathematical model that enables simulation of decision outcomes is provided. The calculations in this document are based on random draws from the distributions in the input table Input_CBA.csv. Explanations for code chunks are not part of the actual simulation. The following code descriptions guide readers through the process of code development.

A function called Simulation is generated to evaluate the decision options in decisionSupport (Luedeling et al., 2021). Within this function the Cost_Benefit function represents the code to compute the costs and benefits for a monitoring strategy over a particular time period. The cashflow and the Net Present Value (NPV) are calculated for the three monitoring strategies by wrapping the Simulation function around the Cost_Benefit function. The following calculations show how the functions are coded (see the entire function below).

```
Simulation <- function(){
  Cost_Benefit <- function(n_years, # number of years to run the simulation
                            highrisk_year, # Years with a high risk of fungal
                            infections
                            CV, # coefficient indicating variation into a
                            time series
                            area, # area of the heather production system
                            sample_costs, # costs per lab sample
                            value_of_discarded_plant, # value of a sampled
                            and then discarded heather plant
                            plant_value_of_A1_quality, # plant value of
                            marketable heather plant that would not have
                            achieved high quality without the respective
                            monitoring strategy.
                            Initial_investment, # Mandatory investment in the
                            first year to start then respective monitoring
                            approach
                            additional_investment, # Occurring investments is
                            the following years
                            labor_costs, # Labor costs to conduct monitoring
                            post_processing_costs, # Data processing of
                            acquired data
                            sample_number, # Number of lab samples
                            additional_saved_plants, # number of heather
                            plants that would not have achieved high
                            marketable quality without the respective
                            monitoring strategy.
                            adjustment_sample_size, # Adjustment to increase
                            sample size in high risk years
                            increased_resource_use_costs, # Costs for
                            increased resource use to protect heather plants
                            in high risk years
                            resource_savings # Monetary savings for reduced
                            resource use in normal risk years due to the
                            respective monitoring strategy
  ){
```

First the labor costs per area are defined. Note that the vv function from the decisionSupport package (Luedeling et al., 2021) is applied to introduce variability into the time series. This function produces a series of n values drawn from a normal distribution, with a mean corresponding to the specified input value (here labor_costs) and a coefficient of variation corresponding to the value of CV.

```
yearly_labor_costs_per_ha <- vv(labor_costs, CV, n = n_years)
```

Then the number of samples per hectare are defined.

```
samples_per_ha <- round(vv(sample_number, CV, n = n_years) *
                  (1+highrisk_year * (vv(adjustment_sample_size, CV,
                  n = n_years))), digits = 0)
```

The sample costs per hectare are defined and the value of discarded plants per hectare is defined for each year.

```
  sample_costs_per_ha <- samples_per_ha * sample_costs

  discarded_plant_value_per_ha <- samples_per_ha *
                                  vv(value_of_discarded_plant, CV,
                                  n = n_years)
```

The total labor and sample costs are defined for the whole production system.

```
  labor_and_sample_cost <- (yearly_labor_costs_per_ha +
                            sample_costs_per_ha +
                            discarded_plant_value_per_ha) * area
```

The initial investment of the first year and additional investments (maintenance costs) of the following years are defined. Then the labor and sample costs, the data post processing-costs, the initial investment and the additional investments are summed up. The result is defined as the total costs.

```
  total_cost <- labor_and_sample_cost +
                vv(post_processing_costs, CV, n = n_years) +
                c(Initial_investment, rep(0,n_years-1)) +
                c(0,vv(additional_investment, CV, n = n_years-1))
```

Benefits of monitoring strategies consist of resource savings that are achieved in normal-risk years and the value of saved plants in high-risk years.

In normal-risk years the number of fungicide applications can be reduced to some extent, because more monitoring increases knowledge about plant health status in the field. Therefore, resource savings can be achieved in these years.

Although monitoring allows for resource savings in normal-risk years, no resource savings are achieved in high-risk years because a higher number of pesticide applications is required. However, thanks to more precise knowledge about the spatial distribution of plant vitality producers are able to protect the quality of more plants, which are safely cultivated until they are sold.

The resource savings in normal-risk years and the value of saved high-quality plants in high-risk years are defined. These monetary values are summed up and the result is defined as the total benefits.

```
    resource_savings <- vv(resource_savings, CV, n = n_years) * area *
                        (1-highrisk_year)

    value_of_high_quality_plants <- vv(additional_saved_plants, CV,
                                       n = n_years)*
                                       plant_value_of_A1_quality *
                                       area * highrisk_year

    total_benefits <- resource_savings + value_of_high_quality_plants
```

In the return command the output of the Cost_Benefit function is defined as cashflow.

```
 return(cashflow = total_benefits - total_cost)}
```

High-risk years can increase sample size and resource use. Occurrence of high-risk years is defined using the chance_event function from decisionSupport (Luedeling et al., 2021). The chance of a high-risk year occurring (chance_high_risk) is defined in the input table as a probability of 40% to 60%, reflecting the probability of risky weather.

```
  highrisk_year <- chance_event(chance_high_risk, n = n_years)
```

The Cost_Benefit function is simulated for standard monitoring (Baseline) and the result is defined as 'cashflow_B'.

```
   cashflow_B <- Cost_Benefit(n_years = n_years,
                              highrisk_year = highrisk_year,
                              CV = var_CV,
                              area = production_area,
                              sample_costs = lab_costs_per_sample,
                              value_of_discarded_plant =
                              plant_value_of_discarded_plant,
                              plant_value_of_A1_quality =
                              plant_value_of_A1_quality,
                              Initial_investment = Initial_investment_B ,
                              additional_investment =
                              additional_investment_B ,
                              labor_costs = labor_costs_B,
                              post_processing_costs =
                              post_processing_costs_B,
                              sample_number = sample_number_B,
                              additional_saved_plants =
                              Number_of_saved_high_quality_plants_B,
                              adjustment_sample_size =
                              adjustment_sample_size_B,
                              resource_savings = resource_savings_B)
```

The Net Present Value is calculated for 'Baseline' monitoring.

```
  NPV_B <- discount(cashflow_B, discount_rate, calculate_NPV = TRUE)
```

The Cost_Benefit function is run for more intense monitoring (Improved) and the result is defined as 'cashflow_I'.

```
cashflow_I <- Cost_Benefit(n_years = n_years,
                            highrisk_year = highrisk_year,
                            CV = var_CV,
                            area = production_area,
                            sample_costs = lab_costs_per_sample,
                            value_of_discarded_plant =
                            plant_value_of_discarded_plant,
                            plant_value_of_A1_quality =
                            plant_value_of_A1_quality,
                            Initial_investment = Initial_investment_I,
                            additional_investment =
                            additional_investment_I ,
                            labor_costs = labor_costs_I,
                            post_processing_costs =
                            post_processing_costs_I,
                            sample_number = sample_number_I,
                            additional_saved_plants =
                            Number_of_saved_high_quality_plants_I,
                            adjustment_sample_size =
                            adjustment_sample_size_I,
                            resource_savings = resource_savings_I)
```

The Net Present Value is calculated for 'Improved' monitoring.

```
NPV_I <- discount(cashflow_I, discount_rate, calculate_NPV = TRUE)
```

The Net Present Value is calculated for the decision to apply 'Improved' instead of 'Baseline' monitoring.

```
comp_NPV_IB <- NPV_I - NPV_B
```

The Cost_Benefit function is run for monitoring with sensor technology (Sensor) and the result is defined as 'cashflow_S'.

```
cashflow_S  <- Cost_Benefit(n_years = n_years,
                            highrisk_year = highrisk_year,
                            CV = var_CV,
                            area = production_area,
                            sample_costs = lab_costs_per_sample,
                            value_of_discarded_plant =
                            plant_value_of_discarded_plant,
                            plant_value_of_A1_quality =
                            plant_value_of_A1_quality,
                            Initial_investment = Initial_investment_S,
                            additional_investment =
                            additional_investment_S,
                            labor_costs = labor_costs_S,
                            post_processing_costs =
                            post_processing_costs_S,
```

```
                              sample_number = sample_number_S,
                              additional_saved_plants =
                              Number_of_saved_high_quality_plants_S,
                              adjustment_sample_size =
                              adjustment_sample_size_S,
                              resource_savings = resource_savings_S)
```

The Net Present Value is calculated for 'Sensor' monitoring.

```
NPV_S <- discount(cashflow_S, discount_rate, calculate_NPV = TRUE)
```

The Net Present Value is calculated for the decision to apply 'Sensor' instead of 'Baseline' monitoring.

```
  comp_NPV_SB <- NPV_S - NPV_B
```

At the end of the Simulation function all calculated outcomes are listed.

```
  return(list(cashflow_B = cashflow_B,
              NPV_B = NPV_B,
              cashflow_I = cashflow_I,
              NPV_I = NPV_I,
              cashflow_S = cashflow_S,
              NPV_S = NPV_S,
              comp_NPV_IB = comp_NPV_IB,
              comp_NPV_SB = comp_NPV_SB))
}
```

The legend file, Monte Carlo results folder, and EVPI results folder are defined.

```
legend_file <- "Legend_CBA.csv"
MC_Results_folder <- "MC_Results_CBA"
EVPI_Results_folder <- "EVPI_Results_CBA"
```

The decisionSupport function is used to run the Monte Carlo simulation.

```
decisionSupport(inputFilePath = input_table,
                outputPath = MC_Results_folder,
                welfareFunction = Simulation,
                write_table = TRUE,
                numberOfModelRuns = 10000,
                functionSyntax = "plainNames")
```

All outcome variables are produced with the decisionSupport function. The Monte Carlo Simulation results are stored in the MC_file and generate the EVPI files.

```
MC_file <- read.csv(paste(MC_Results_folder,"/mcSimulationResults.csv",
           sep = ""))

MC_file_without_cashflow <- select(MC_file, -c(1, starts_with("cashflow")))
multi_EVPI(mc = MC_file_without_cashflow,first_out_var = "NPV_B",
           write_table = TRUE,outfolder = EVPI_Results_folder)
```

```
welfare_summary <- read.csv(paste(MC_Results_folder,
                   "/welfareDecisionSummary.csv",sep = ""))
```

R function: Simulation

Here the code is shown in one piece for an overview of all calculations.

```
Simulation <- function(){

  Cost_Benefit <- function(n_years,
                           highrisk_year,
                           CV,
                           area,
                           sample_costs,
                           value_of_discarded_plant,
                           plant_value_of_A1_quality,
                           Initial_investment,
                           additional_investment,
                           labor_costs,
                           post_processing_costs,
                           sample_number,
                           additional_saved_plants,
                           adjustment_sample_size,
                           increased_resource_use_costs,
                           resource_savings){

    yearly_labor_costs_per_ha <- vv(labor_costs, CV, n = n_years)

    samples_per_ha <- round(vv(sample_number, CV, n = n_years) *
      (1+highrisk_year * (vv(adjustment_sample_size, CV, n = n_years))),
      digits = 0)

    sample_costs_per_ha <- samples_per_ha * sample_costs

    discarded_plant_value_per_ha <- samples_per_ha *
                                    vv(value_of_discarded_plant, CV,
                                    n = n_years)

    labor_and_sample_cost <- (yearly_labor_costs_per_ha +
                              sample_costs_per_ha +
                              discarded_plant_value_per_ha) * area

    total_cost <- labor_and_sample_cost +
                  vv(post_processing_costs, CV, n = n_years) +
                  c(Initial_investment, rep(0,n_years-1)) +
                  c(0,vv(additional_investment, CV, n = n_years-1))

    resource_savings <- vv(resource_savings, CV, n = n_years) * area *
                        (1-highrisk_year)
```

```
  value_of_high_quality_plants <- vv(additional_saved_plants, CV,
                                     n = n_years) *
                                     plant_value_of_A1_quality * area *
                                     highrisk_year

  total_benefits <- resource_savings + value_of_high_quality_plants

  return(cashflow = total_benefits - total_cost)}

highrisk_year <- chance_event(chance_high_risk, n = n_years)

cashflow_B <- Cost_Benefit(n_years = n_years,
                            highrisk_year = highrisk_year,
                            CV = var_CV,
                            area = production_area,
                            sample_costs = lab_costs_per_sample,
                            value_of_discarded_plant =
                            plant_value_of_discarded_plant,
                            plant_value_of_A1_quality =
                            plant_value_of_A1_quality,
                            Initial_investment = Initial_investment_B,
                            additional_investment =
                            additional_investment_B,
                            labor_costs = labor_costs_B,
                            post_processing_costs =
                            post_processing_costs_B,
                            sample_number = sample_number_B,
                            additional_saved_plants =
                            Number_of_saved_high_quality_plants_B,
                            adjustment_sample_size =
                            adjustment_sample_size_B,
                            resource_savings = resource_savings_B)

NPV_B <- discount(cashflow_B, discount_rate, calculate_NPV = TRUE)

cashflow_I <- Cost_Benefit(n_years = n_years,
                            highrisk_year = highrisk_year,
                            CV = var_CV,
                            area = production_area,
                            sample_costs = lab_costs_per_sample,
                            value_of_discarded_plant =
                            plant_value_of_discarded_plant,
                            plant_value_of_A1_quality =
                            plant_value_of_A1_quality,
                            Initial_investment = Initial_investment_I,
                            additional_investment =
                            additional_investment_I ,
                            labor_costs = labor_costs_I,
                            post_processing_costs =
```

```
                              post_processing_costs_I,
                              sample_number = sample_number_I,
                              additional_saved_plants =
                              Number_of_saved_high_quality_plants_I,
                              adjustment_sample_size =
                              adjustment_sample_size_I,
                              resource_savings = resource_savings_I)

  NPV_I <- discount(cashflow_I, discount_rate, calculate_NPV = TRUE)

  comp_NPV_IB <- NPV_I - NPV_B

  cashflow_S  <- Cost_Benefit(n_years = n_years,
                              highrisk_year = highrisk_year,
                              CV = var_CV,
                              area = production_area,
                              sample_costs = lab_costs _per_sample,
                              value_of_discarded_plant =
                              plant_value_of_discarded_plant,
                              plant_value_of_A1_quality =
                              plant_value_of_A1_quality,
                              Initial_investment = Initial_investment_S,
                              additional_investment =
                              additional_investment_S ,
                              labor_costs = labor_costs_S,
                              post_processing_costs =
                              post_processing_costs_S,
                              sample_number = sample_number_S,
                              additional_saved_plants =
                              Number_of_saved_high_quality_plants_S,
                              adjustment_sample_size =
                              adjustment_sample_size_S,
                              resource_savings = resource_savings_S)

  NPV_S <- discount(cashflow_S, discount_rate, calculate_NPV = TRUE)

  comp_NPV_SB <- NPV_S - NPV_B

  return(list(cashflow_B = cashflow_B,
              NPV_B = NPV_B,
              cashflow_I = cashflow_I,
              NPV_I = NPV_I,
              cashflow_S = cashflow_S,
              NPV_S = NPV_S,
              comp_NPV_IB = comp_NPV_IB,
              comp_NPV_SB = comp_NPV_SB))
}

decisionSupport(inputFilePath = input_table,
                outputPath = MC_results_folder,
```

```
                welfareFunction = Simulation,
                write_table = TRUE,
                numberOfModelRuns = 10000,
                functionSyntax = "plainNames")

MC_file <- read.csv(paste(MC_Results_folder,"/mcSimulationResults.csv",
           sep = ""))

MC_file_without_cashflow <- select(MC_file, -c(1, starts_with("cashflow")))
multi_EVPI(mc = MC_file_without_cashflow,first_out_var = "NPV_B",
           write_table = TRUE,outfolder = EVPI_Results_folder)

welfare_summary <- read.csv(paste(MC_Results_folder,
                  "/welfareDecisionSummary.csv",sep = ""))
```

Calculation of the expected utility

The expected utility is calculated for different monitoring strategies. First, the simulation results are loaded into the R environment. The select function from the dplyr package (Wickham et al., 2019a) is used to select the simulated Net Present Values for each monitoring strategy.

```
data_B <- read.csv("mcSimulationResults.csv")
data_B <- dplyr::select(data_B, starts_with("NPV_B")) %>%
          stack(drop=FALSE)
data_B$values <- as.numeric(data_B$values)

data_I <- read.csv("mcSimulationResults.csv")
data_I <- dplyr::select(data_I, starts_with("NPV_I")) %>%
          stack(drop=FALSE)
data_I$values <- as.numeric(data_I$values)

data_S <- read.csv("mcSimulationResults.csv")
data_S <- dplyr::select(data_S, starts_with("NPV_S")) %>%
          stack(drop=FALSE)
data_S$values <- as.numeric(data_S$values)
```

Then the risk premium calculation is started for the monitoring strategies.

```
risk_premium_B <-(1/2)*0.0001*var(data_B$values)

risk_premium_I <-(1/2)*0.0001*var(data_I$values)

risk_premium_S <-(1/2)*0.0001*var(data_S$values)
```

The semivariance is calculated for the monitoring strategies.

```
for(z in 1 : 10000){
  data_B$NewColumn[z] <- ifelse(min((data_B$values[z]- mean(data_B$values)),
                         0) < 0,(data_B$values[z]- mean(data_B$values))^2, 0)
}
```

```r
Semivaricance_B <- mean(data_B$NewColumn)

for(z in 1 : 10000){
  data_I$NewColumn[z] <- ifelse(min((data_I$values[z]- mean(data_I$values)),
                        0) < 0,(data_I$values[z]- mean(data_I$values))^2, 0)
}
Semivaricance_I <- mean(data_I$NewColumn)

for(z in 1 : 10000){
  data_S$NewColumn[z] <- ifelse(min((data_S$values[z]- mean(data_S$values)),
                        0) < 0,(data_S$values[z]- mean(data_S$values))^2, 0)
}
Semivaricance_S <- mean(data_S$NewColumn)
```

The certainty equivalents and the risk aversion coefficients are computed and the results are stored in a data frame called risk_aversion_data.

```r
for(r in c(-1e-01, -1e-02, -1e-03, -1e-04, -1e-05, -1e-06, 0,
           1e-06,  1e-05, 1e-04, 1e-03, 1e-02, 1e-01)){
  Certainty_equivalent_B_ <- mean(data_B$values) - Semivaricance_B * r
  assign(paste("CE_B_", r, sep = ""), Certainty_equivalent_B_)

  Certainty_equivalent_I_ <- mean(data_I$values) - Semivaricance_I * r
  assign(paste("CE_I_", r, sep = ""), Certainty_equivalent_I_)

  Certainty_equivalent_S_ <- mean(data_S$values) - Semivaricance_S * r
  assign(paste("CE_S_", r, sep = ""), Certainty_equivalent_S_)
}

Certainty_equivalent <- c(`CE_B_-0.1`,`CE_B_-0.01`,`CE_B_-0.001`,
                          `CE_B_-1e-04`,`CE_B_-1e-05`,`CE_B_-1e-06`,
                          `CE_B_0`,`CE_B_1e-06`,`CE_B_1e-05`,`CE_B_1e-04`,
                          `CE_B_0.001`,`CE_B_0.01`,`CE_B_0.1`,`CE_I_-0.1`,
                          `CE_I_-0.01`,`CE_I_-0.001`,`CE_I_-1e-04`,
                          `CE_I_-1e-05`,`CE_I_-1e-06`,`CE_I_0`,`CE_I_1e-06`,
                          `CE_I_1e-05`,`CE_I_1e-04`,`CE_I_0.001`,`CE_I_0.01`,
                          `CE_I_0.1`,`CE_S_-0.1`,`CE_S_-0.01`,`CE_S_-0.001`,
                          `CE_S_-1e-04`,`CE_S_-1e-05`,`CE_S_-1e-06`,`CE_S_0`,
                          `CE_S_1e-06`,`CE_S_1e-05`,`CE_S_1e-04`,
                          `CE_S_0.001`, `CE_S_0.01`, `CE_S_0.1`)

monitoring_type <- c(rep("Baseline", 13),rep("Improved", 13),rep("Sensor",
                   13))

risk_aversion_coefficient <- c(-1e-01, -1e-02, -1e-03, -1e-04, -1e-05,
                              -1e-06, 0, 1e-06, 1e-05, 1e-04, 1e-03, 1e-02,
                               1e-01)

options(scipen = 999)
risk_aversion_data <- data.frame(Certainty_equivalent,
                      risk_aversion_coefficient, monitoring_type)
```

References

Henry, L., Wickham, H., & Chang, W. (2019). *Ggstance: Horizontal ’ggplot2’ Components*. https://CRAN.R-project.org/package=ggstance

Kassambara, A. (2019). *Ggpubr: ’Ggplot2’ Based Publication Ready Plots*. https://CRAN.R-project.org/package=ggpubr

Luedeling, E., Goehring, L., Schiffers, K., Whitney, C., & Fernandez, E. (2021). *decisionSupport: Quantitative Support of Decision Making under Uncertainty*. http://www.worldagroforestry.org/

Wickham, H. (2016). *Ggplot2: Elegant Graphics for Data Analysis*. Springer-Verlag New York. https://ggplot2.tidyverse.org

Wickham, H., François, R., Henry, L., & Müller, K. (2019a). *Dplyr: A Grammar of Data Manipulation*. https://CRAN.R-project.org/package=dplyr

Wickham, H., Hester, J., & Chang, W. (2019b). *Devtools: Tools to Make Developing R Packages Easier*. https://CRAN.R-project.org/package=devtools

Xie, Y. (2019a). *Knitr: A General-Purpose Package for Dynamic Report Generation in R*.

Xie, Y. (2019b). *Bookdown: Authoring Books and Technical Documents with R Markdown*.

Zhu, H. (2019). *kableExtra: Construct Complex Table with ’kable’ and Pipe Syntax*. https://CRAN.R-project.org/package=kableExtra
